# Genetic Risk Factors and Clinical Implications of Glaucoma in the Saudi Population: A Review

**DOI:** 10.3390/ijms27083506

**Published:** 2026-04-14

**Authors:** Abdullah Faisal Alotaibi, Lojain Mohammed A. Maawadh, Mohammed Naji Obaid Almutairi, Syed Hameed, Rizwan Malik, Khaled K. Abu-Amero

**Affiliations:** 1 Glaucoma Division, King Khaled Eye Specialist Hospital and Research Center, Riyadh 11462, Saudi Arabia; abdullalotaibi147@gmail.com; 2 College of Medicine, Imam Abdulrahman Bin Faisal University, Dammam 31441, Saudi Arabia; lojainmwdh@gmail.com; 3 College of Medicine, King Saud bin Abdulaziz University for Health Sciences, Riyadh 11481, Saudi Arabia; m.alesemer82@gmail.com; 4 Research Department, King Khaled Eye Specialist Hospital and Research Center, Riyadh 11462, Saudi Arabia; syhameed@kkesh.med.sa (S.H.); rmalik@kkesh.med.sa (R.M.)

**Keywords:** glaucoma, genetics, Saudi Arabia, consanguinity, risk factors, CYP1B1, APOE, personalized medicine

## Abstract

Most glaucoma genetic data derive from European and East Asian cohorts, leaving high-consanguinity Middle Eastern populations under-characterized. This review synthesizes 33 Saudi-specific genetic studies (2014–2024, >9000 participants) to define a population-level glaucoma genetic architecture that diverges substantially from global models and carries direct precision medicine implications. Three findings distinguish the Saudi landscape. First, *CYP1B1* functions as the dominant causal gene across both primary congenital glaucoma (PCG) and juvenile-onset open-angle glaucoma (JOAG), accounting for 76–86% of cases, with two founder alleles, p.G61E (penetrance 87.7%) and p.R469W (penetrance 93%), driving severe, early-onset phenotypes. Critically, *MYOC* and *LTBP2*, the primary JOAG genes in other populations, carry no pathogenic variants in Saudi cohorts, rendering standard multi-ethnic gene panels inadequate for this population. Second, adult-onset glaucoma follows a distinct polygenic architecture where *APOE* ε2 confers a near five-fold risk for primary angle-closure glaucoma (OR = 4.82), an effect absent or inconsistent in global datasets, and *NOS3* variants associate with primary open-angle glaucoma specifically in men, a sex-stratified signal unreported outside Saudi cohorts. The *MTHFR* T/T genotype, common in European and Asian POAG patients, is entirely absent locally, indicating population-specific allelic distributions that alter folate-metabolism-related optic nerve susceptibility. Third, *ACVR1* rs12997 associates across POAG, PACG, and pseudoexfoliation glaucoma (PXG), positioning BMP/TGF-β signaling as a shared mechanistic pathway spanning multiple subtypes. These findings argue for Saudi-specific genetic panels, *CYP1B1*-centered cascade testing in consanguineous families, and polygenic risk models incorporating local allele frequencies rather than globally derived weights.

## 1. Introduction

Glaucoma is a progressive optic neuropathy marked by loss of retinal ganglion cells, degeneration of the optic nerve, thinning of the retinal nerve fiber layer, and progressive excavation of the optic disc, culminating in visual field loss and potential blindness. The disorder poses a major public-health burden worldwide and shows substantial variation in prevalence and causes across ethnic groups [1]. Understanding the genetic contributors to glaucoma is therefore essential, particularly in populations with distinct demographic features such as Saudi Arabia, where inherited predispositions may differ from those reported elsewhere [2].

Globally, glaucoma affects an estimated 80 million people, a figure projected to rise to about 111 million by 2040 [3]. Elevated intraocular pressure (IOP) is a major driver, but the disease presents in several clinically and epidemiologically distinct subtypes: POAG (typically insidious), PACG (often related to anterior-segment anatomy and acute IOP elevation), juvenile-onset open-angle glaucoma (JOAG, onset before age 40 and frequently monogenic) [4], and pseudoexfoliation glaucoma (PXG, associated with fibrillar extracellular deposits) [5]. Primary congenital glaucoma (PCG) is a particularly important cause of childhood blindness in Saudi Arabia, where incidence estimates (approximately 1 in 2500 live births) exceed global averages and are thought to reflect, in part, high consanguinity rates [6].

In Saudi Arabia, glaucoma accounts for a substantial proportion of visual disability [7]. Consanguineous marriage is common in Saudi Arabia and increases the probability of homozygous pathogenic variants; reported consanguinity rates range from roughly 52% to 58%, and can be higher in some rural areas (80%), amplifying recessive alleles such as those in *CYP1B1* observed in JOAG and PCG cohorts [8]. All reported results here are related to the Saudi glaucoma patients.

National estimates place overall glaucoma prevalence near 5.1%, but the relative frequency of subtypes varies by region and by study setting: for example, PACG predominated in a Riyadh tertiary series (46.6%) [9], whereas POAG was more common in an Al-Ahsa cohort (60%) [10]. Other regional reports show differing subtype mixes: POAG (27.7%) and secondary glaucoma (26.7%) are the most frequent in the Eastern Province, while PCACG (46.6%) and POAG (25.6%) are the most frequent in Qassim [11,12]. PCG, as an autosomal recessive disease, may be more prevalent in the south as consanguinity there can reach up 60% of all newlywed couples and in areas like Samtah, a peak rate of 80.6% [13]. These regional differences reflect referral patterns and methodological variation and point to the need for large, population-based surveys to define the true epidemiology.

Genetic investigations have linked multiple genes and single-nucleotide polymorphisms to the principal clinical forms of glaucoma, primary open-angle glaucoma (POAG) and PACG, underscoring the condition’s heterogeneity [2]. In Saudi cohorts, pathogenic variants in *CYP1B1* have been repeatedly implicated in juvenile-onset open-angle glaucoma, supporting the potential value of targeted genetic testing in high-risk families for earlier diagnosis and management [14]. Secondary glaucoma subtypes are also relevant locally: for example, neovascular glaucoma has been reported at rates up to 13% in some Saudi series, with diabetes mellitus present in the majority of affected patients (88%) in one retrospective study [15].

This review incorporated publications from 2010 to December 2025, presented genetic or polymorphism data from Saudi cohorts, and addressed at least one of five glaucoma subtypes (POAG, PACG, PXG, JOAG, PCG). We omitted studies that did not involve a Saudi cohort. This narrative review examines how population genetics and consanguinity shape glaucoma risk in Saudi Arabia, summarizes genetic studies performed in Saudi cohorts, evaluates the clinical relevance of reported polymorphisms, and identifies priorities for future research and translation.

## 2. Genetic Aspects of Glaucoma in the Saudi Population

### 2.1. Overview of Study Characteristics

A total of 33 case–control studies were conducted between 2014 and 2024, comprising more than 9000 Saudi participants, illustrating various glaucoma subtypes, POAG, PACG, PXG, JOAG, and PCG, and their association with specific genetic polymorphisms in the Saudi population. The evidence concentrates on causative genes in congenital/juvenile disease (*CYP1B1*), core PXG risk genes (*LOXL1*), and protective microRNA biogenesis signals (DICER1), alongside polygenic contributors in adult glaucoma (*APOE*, *ACVR1*, *NOS3*, *MTHFR*, *SMOC2*).

### 2.2. Congenital and Juvenile Onset Glaucoma

In the Saudi population, early-onset glaucoma frequently exhibits a Mendelian inheritance pattern, largely driven by the high prevalence of consanguinity. Genes and their associated variants with glaucoma subtypes, along with penetrance, are summarized in Table 1.

#### 2.2.1. Primary Congenital Glaucoma (PCG)

Primary congenital glaucoma is caused by trabecular meshwork dysgenesis, leading to elevated intraocular pressure and eye injury. Mutations in *CYP1B1* constitute the predominant identified genetic etiology. Nonetheless, significant inquiries regarding the penetrance of *CYP1B1*-associated congenital glaucoma remain unresolved. Additionally, alterations in other genes have been reported, but their precise contribution and potential genetic interaction, if any, with *CYP1B1*-mutations remain inadequately investigated.

A cross multiple Saudi PCG cohorts, pathogenic variants in *CYP1B1* were detected in the majority of cases, confirming it as the predominant causative gene [20]. One Saudi study reported a mutation in 79.4% of PCG patients [16], while larger cohorts reported variants in up to 80.8% [17] and 76% [18] of cases. The majority of PCG cases in Saudi Arabia exhibiting mutations in the cytochrome P450 family 1 subfamily B member 1.

(*CYP1B1*) gene demonstrates an autosomal recessive inheritance pattern, attributable to the prevalent consanguinity in the Kingdom [21]. No mutations in *LTBP2*, *MYOC* or *TEK* were identified in the Saudi population. The primary gene altered in the PCG-Saudi patients is *CYP1B1*. The recurrent alleles p.G61E and p.R469W in the *CYP1B1* gene were the most frequently observed. As detailed in Table 1, p.G61E penetrance was estimated at 87.7% and p.R469W at 93%. The homozygous null genotypes and the presence of p.G61E or p.R469W were associated with more severe clinical phenotypes, including earlier age at onset, higher indices of corneal haze, and larger corneal diameters [16,18]. A subset of PCG cases with negative *CYP1B1* mutations yielded candidate genes including *SLC4A4*, *SLC4A11*, *CPAMD8*, *KERA*, and *ADAM9* [17], but with no functional studies linking those genes to PCG.

#### 2.2.2. Juvenile Open-Angle Glaucoma (JOAG)

JOAG in Saudi Arabia is an uncommon yet serious, frequently genetic, early-onset variant of glaucoma (mostly affecting individuals aged 3–35) marked by elevated intraocular pressure and a substantial risk of visual field deterioration. It constitutes roughly 1.8% to 2% of glaucoma cases in Saudi higher institutions. Its severity frequently necessitates prompt surgical intervention and continuous monitoring, with research demonstrating elevated rates of myopia (93%) and a significant familial predisposition (51.1%) among patients. In the Saudi JOAG cohort, *CYP1B1* variants were present in 85.7% of patients [14]. The predominant mutation was p.G61E, most often homozygous. This high frequency contrasts with other populations where *MYOC* is more common; sequencing of *MYOC* and *LTBP2* in Saudi JOAG patients identified no pathogenic variants [14]. This suggests that JOAG in this population often represents a delayed-onset phenotype of the same genetic defects causing PCG.

### 2.3. Adult-Onset Glaucoma

Adult-onset forms of glaucoma in Saudi Arabia (POAG, PACG, PXG) display a complex genetic architecture involving multiple susceptibility alleles rather than a single causative gene. The statistical associations for these risk factors are detailed in Table 2.

#### 2.3.1. Primary Open-Angle Glaucoma (POAG)

POAG is one of the most prevalent subtypes of glaucoma globally, including in the Middle East [9]. Numerous genetic and hereditary factors, including family history, age, intraocular pressure (IOP), and ancestry, are recognized risk factors for POAG [33]. The ethnic propensity and familial tendency strongly indicate the role of genetic factors in the development of POAG [33]. Previous studies have shown that common genes associated with familial POAG like *OPTN* and *MYOC* lack mutations in the Saudi population [34].

In the recent past, genome-wide association studies (GWAS) have identified several significant associations of POAG or its endophenotypic traits with single nucleotide polymorphisms (SNPs) in different genes/loci [33,35]. These include the rs35934224 in the thioredoxin reductase 2 (*TXNRD2*) genomic regions [36], and rs6478746 on chromosome 9 near LIM homeobox transcription factor 1 beta (*LMX1B*) and an uncharacterized RNA gene, *LOC105376277* identified among the POAG patients of European ancestry [37]. The rs10483727 “T” allele of the *SIX1/SIX6* locus was consistently associated with POAG in Saudi cases (OR = 1.7) [22]. *NOS3*: Polymorphisms rs2070744 and rs1799983 were significantly associated with POAG, particularly in men, indicating a sex-specific risk linked to endothelial dysfunction [23]. *MTHFR*: The C677T polymorphism showed a unique profile; the C/T genotype was more common in cases (OR = 1.60), while the C/C genotype was protective. The T/T genotype was notably absent in the Saudi population [24]. *ACVR1*: The rs12997 variant (G allele) showed a modest association (OR = 1.39) with POAG [25]. All our POAG patients had inclusion criteria, which is based on optic nerve findings, open angles, and VF loss with no secondary cause. There was no specification of the IOP threshold for case inclusion, meaning our cohorts almost certainly contain a mix of NTG and HTG without reporting the proportion.

#### 2.3.2. Primary Angle-Closure Glaucoma (PACG)

PACG is a multifaceted, polygenic disorder with significant hereditary influences, especially among Asian populations, influenced by anatomical risk factors such as shallow anterior chambers and dense ocular tissues. Genome-Wide Association Studies (GWAS) studies have identified various susceptibility loci such as *PLEKHA7, COL11A1, PCMTD1-ST18, EPDR1, CHAT, GLIS3, FERMT2,* and *DPM2-FAM102A*, which were implicated in cell adhesion and ocular development. These SNPs, risk factors, are located in genes like *PLEKHA7* (Engaged in cell adhesion at the zonula adherens); *COL11A1* (Associated with collagen synthesis and structural integrity); *FERMT2* & *EPDR1* genes involved in cell signaling and extracellular matrix regulation and *CHAT*, which encodes choline O-acetyltransferase, pertinent to neurotransmitter [38]. In addition, the ε2 allele of the *APOE* gene is a strong risk factor. Carriers of ε2 had a 4.8-fold increased risk (OR = 4.82) [26]. The rs12997 G/G genotype in the *ACVR1* gene was associated with >2-fold increased risk, particularly in women [27]. The rs13208776 G/A genotype in the *SMOC2* gene was significantly more frequent in PACG cases (OR = 2.16) [28]. The c.47T > C (rs4880) polymorphism in the *SOD2* gene was not associated with disease susceptibility, but was significantly correlated with severity markers, including earlier age at onset and poorer visual acuity [29].

#### 2.3.3. Pseudoexfoliation Glaucoma (PXG)

PXG is a complex, age-related, secondary open-angle glaucoma primarily linked to mutations in the *LOXL1* (lysyl oxidase-like 1) gene on chromosome 15q24.1. This gene is important for making elastin and breaking down extracellular matrix. If it doesn’t work, fibrillar material builds up and stops water from flowing out. SNPs in *LOXL1* are significantly associated with the initiation of pseudoexfoliation syndrome (PXS) and its subsequent advancement to pseudoexfoliative glaucoma (PXG) across diverse ethnicities. While *LOXL1* is a crucial factor, risk alleles vary across populations; for example, high-risk SNPs in Scandinavian cohorts may offer protection in different groups. Not all individuals possessing high-risk *LOXL1* mutations exhibit the disease, indicating the influence of supplementary genetic, epigenetic (e.g., RNA methylation), or environmental factors. Other candidate genes, like *CACNA1A* (which is linked to PXS in some groups) and APOE, may also affect the risk. Epigenetic modifications, encompassing DNA methylation and non-coding RNA, are believed to play a role in disease development. PXG is considered a systemic disease, with genetic predisposition usually defined by complex, multifactorial inheritance rather than a simple Mendelian pattern. In the *LOXL1* gene: the global risk alleles (G of rs1048661 and rs3825942) are confirmed in Saudis (*p* = 0.0056 and *p* = 0.000005). Additionally, two novel non-synonymous SNPs (D484E and Y559D) were detected exclusively in Saudi patients [31]. A protective association was found in the *DICER1* gene (rs3742330) G allele and the G/A haplotype across *DICER1*/*DROSHA* were protective against PXG (OR = 0.38) [30]. As for the *ACVR1*, a weaker signal was observed for rs12997, with the G/G genotype trending toward increased risk (OR = 2.04) [27].

### 2.4. Polymorphisms Without Significant Association

Multiple SNPs were examined but revealed no significant association with glaucoma in the Saudi population. These exclusions are essential for distinguishing local genetic architecture from global trends: *TXNRD2* (rs35934224) shows no correlation with PACG, whereas rs6478746 in *LMX1B* [39,40] also exhibits no association with PACG and PFX. The SNPs previously recognized as risk factors in other ethnic groups were not correlated with different forms of glaucoma in the Saudi population. The variations *GAS7* (rs11656696), *TMCO1* (rs7555523), *TLR2*, and *TLR4* were not correlated with primary open-angle glaucoma (POAG) in the Saudi population. Furthermore, the subsequent SNPs exhibited no correlation with POAG. The variants *CDKN2B* (rs1063192) [41] and *ATOH7* (rs1900004) [42], as well as the 1q43 locus variants [43,44], *PLXDC2* [45], *FNDC3B* and *ABCA1*, exhibited no connection with POAG [46].

## 3. Discussion

### 3.1. Population-Specific Trends

The genetic profile of glaucoma in Saudi Arabia departs from patterns reported in many Western and Asian populations, a divergence driven largely by high consanguinity and population-specific allele distributions [16,17,47]. Consanguineous marriage increases homozygosity and thereby amplifies recessive alleles. This demographic feature helps explain the unusually high prevalence of *CYP1B1*-related early-onset glaucoma in Saudi cohorts [16,17,18].

Population surveys confirm that consanguinity is common in Saudi Arabia, with an overall 56%, with 60% first cousin marriages. This pattern is associated with increased prevalence of certain recessive disorders and relevant to glaucoma genetics [47]. Within this demographic context, PCG stands out, with multiple Saudi series reporting *CYP1B1* mutation rates between 76% and 85%, with recurrent founder alleles p.G61E and p.R469W showing high penetrance and strong links to severe, early onset phenotypes [16,17,18]. However, p.R368H exhibits low penetrance and has been reclassified as likely benign in larger family studies [29]. Several novel *CYP1B1* alleles, including p.E229K, p.H279L, and p.R355X, have been described in Saudi cohorts, underscoring ongoing mutational heterogeneity and the need for comprehensive sequencing in unsolved cases [17,18].

A minority of PCG patients remain *CYP1B1* negative, where cases have produced candidate loci, including *SLC4A4*, *SLC4A11*, *CPAMD8*, *KERA*, *ADAM9*, and provisional signals (*BCO2*, *TULP2*, *DGKQ*) that require replication and functional follow-up [17].

In juvenile-onset disease, *CYP1B1* emerges as the dominant causal gene in the Saudi JOAG series, where pathogenic *CYP1B1* variants are found in the majority of cases, with p.G61E the most frequent allele, and no pathogenic *MYOC* or *LTBP2* variants are detected. These observations support targeted genetic testing for *CYP1B1* in early-onset Saudi patients and argue for population-tailored counseling and cascade testing in affected families [14]. *CYP1B1* dysfunction is believed to disrupt early trabecular meshwork development. However, the exact mechanism remains unclear [48]. Mutations in the latent transforming growth factor-beta-binding protein 2 (*LTBP2*) gene have also been associated with PCG but are uncommon [49]. Unlike PCG caused by *CYP1B1*, recessive *LTBP2* mutations are linked to congenital megalocornea and childhood secondary glaucoma resulting from spherophakia and/or ectopia lentis [50].

Juvenile and adult glaucoma in Saudi Arabia occupy opposite ends of the genetic architecture spectrum. Juvenile-onset disease (JOAG and PCG) is effectively monogenic, with *CYP1B1* the causative gene in 76–86% of cases, driven by two high-penetrance founder alleles, p.G61E (87.7%) and p.R469W (93%), both amplified by high consanguinity rates. Adult-onset glaucoma (POAG, PACG, PXG) is polygenic, where no single gene dominates and risk is distributed across susceptibility loci with modest effect sizes: *SIX1/SIX6*, *NOS3*, and *MTHFR* for POAG; *APOE* ε2, *ACVR1*, and *SMOC2* for PACG; *LOXL1* and *FNDC3B* for PXG. The one gene bridging both ends is *ACVR1*, whose rs12997 variant shows consistent, if modest, associations across POAG, PACG, and PXG, implicating BMP/TGF-β signaling as a shared pathophysiological thread. *MYOC* and *LTBP2*, dominant in JOAG in other populations, carry no pathogenic variants in Saudi cohorts, confirming that standard multi-ethnic panels misrepresent local risk. For precision medicine, this stratifies cleanly: early-onset patients warrant single-gene *CYP1B1* sequencing with cascade family testing, while adult patients require polygenic risk profiling acknowledging Saudi-specific allele distributions. *NOS3* sex-stratified effects in POAG and the near-five-fold *APOE* ε2 risk in PACG offer the clearest actionable pharmacogenomic signals in adult disease. *NOS3* variants reduce endothelial nitric oxide bioavailability, impairing autoregulation of optic nerve head perfusion and elevating IOP, but the vascular dysfunction they produce also activates downstream inflammatory cascades, including NF-κB-mediated upregulation of cytokines and endothelial adhesion molecules that promote retinal ganglion cell loss independently of pressure. The sex-stratified association in Saudi POAG men likely reflects androgen-modulated eNOS expression rather than a simple vascular effect, linking hormonal biology to both perfusion failure and neuroinflammatory susceptibility. This dual vascular–immunological mechanism positions *NOS3* as a plausible pharmacogenomic target for nitric oxide-donor or anti-inflammatory trials stratified by both genotype and sex.

Adult-onset glaucoma in Saudis shows a different pattern, with risk that is distributed across multiple susceptibility loci rather than dominated by a single, highly penetrant mutation. For POAG, the *SIX1/SIX6* rs10483727 T allele recurs across studies, and endothelial *NOS3* variants (rs2070744, rs1799983) associate with disease, with stronger effects reported in men [22,23]. *NOS3* genes are associated with an increased risk of POAG, particularly high-tension POAG and cases with specific vascular dysfunction. Variants like T-786C and G894T (Glu298Asp) may alter eNOS function, reducing nitric oxide production, which is crucial for regulating ocular blood flow and reducing intraocular pressure [22,23]. Comparison with large trans-ancestry GWAS shows partial concordance: several loci identified in the GWAS meta-analysis (for example, *SIX1/SIX6*) replicate in Saudi POAG cohorts, whereas strong Saudi signals driven by population structure and consanguinity (notably *CYP1B1* in JOAG/PCG and Saudi-specific *LOXL1* variants in PXG) are absent or attenuated in the global meta-analysis, underscoring the value of cross-ancestry meta-analysis to distinguish universally relevant loci from population-specific causal alleles [51]. Analysis of fine-sectored circumpapillary retinal nerve fiber layer thickness (cpRNFLT) reveals that SIX1/SIX6 polymorphisms, particularly rs33912345, correlate with localized cpRNFL thinning in the inferior area (281.3–303.8°), as reported in Nature. This particular structural thinning is associated with functional parafoveal scotoma in patients with glaucoma [52].

The *MTHFR* C677T genetic variation shows a unique pattern in the Saudi population, with the C/T genotype being more common in patients with POAG, while the C/C genotype seems to offer protection against the disease. The T/T genotype was absent, underscoring a unique allelic distribution in Saudis compared with European and Asian populations, where T/T carriers are more common. This distribution suggests that issues with folate metabolism and elevated homocysteine levels may be linked to optic nerve susceptibility in this group [24]. Additionally, the *ACVR1* rs12997 variant has demonstrated consistent, albeit modest, associations across POAG studies, suggesting that variations in the BMP/TGF-β signaling pathways may play a role in the development of this condition in adults [25,27].

PACG in Saudi cohorts is characterized by notable *APOE* effects, with the ε2 allele and ε2/ε3 heterozygotes conferring a nearly five-fold increased risk, while no consistent link to PXG was observed [26]. These findings contrast with large international datasets (for example, UK Biobank), where *APOE* effects are inconsistent, and they emphasize the value of local genetic studies for risk stratification [33]. APOE participates in lipid metabolism and is synthesized by retinal Müller cells and astrocytes. The association indicates that APOE variations may alter the degeneration of retinal ganglion cells (RGCs) via IOP-independent processes or by influencing lipid metabolism and inflammation, both of which are pertinent to glaucoma etiology [26].

The *SMOC2* variant rs13208776 is also identified as a marker linked to the susceptibility of PACG in Saudi individuals, with the G/A genotype being more common in affected individuals, while the G/G genotype is less frequent. This suggests a role for extracellular matrix pathways in the biology of angle-closure conditions [28]. Importantly, despite these susceptibility alleles, no monogenic causative mutations for PACG have been identified, and the evidence supports a polygenic and multifactorial model for PACG risk in this population [26,27].

PEG replicates global *LOXL1* associations in Saudi patients, where rs1048661 and rs3825942 “G” alleles are enriched in cases, and two non-synonymous variants (D484E, Y559D) have been reported uniquely in Saudi patients, with Y559D predicted to be damaging by in silico tools [31,53]. However, regarding PXA, a protective signal from *DICER1* rs3742330 (G-allele) and a protective G/A haplotype across *DICER1/DROSHA* indicate that microRNA biogenesis may modulate pseudoexfoliation risk and severity. This finding is particularly relevant in Saudi Arabia, where prevalence increases with age [30]. A landmark GWAS study demonstrated that *LOXL1* variants confer an exceptionally high risk of exfoliation glaucoma, with the high-risk haplotype accounting for more than 99% of cases in Icelandic and Swedish populations [53]. LOXL1 (lysyl oxidase-like 1) plays a role in the pathogenesis of PXG by promoting the accumulation of aberrant, excessive elastin-rich fibrillar material, which obstructs ocular drainage structures (trabecular meshwork) and compromises connective tissues (lamina cribrosa). It is regarded as the primary genetic risk factor for the syndrome, functioning through intricate, stage-dependent pathways.

In comparison to other populations datasets, *SIX1/SIX6* (rs10483727) is the one locus that replicates cleanly between Saudi POAG cohorts (OR = 1.7) and the 127-locus trans-ancestry GWAS meta-analysis (Gharahkhani et al., 2021, Nat Commun) [51], confirming it as a genuinely universal POAG signal rather than a population-specific artifact. Everything else diverges considerably. *TXNRD2* (rs35934224) and *LMX1B* (rs6478746), both identified in European POAG GWAS, show no association in Saudi cohorts, indicating that European-derived loci do not transfer reliably to this population. *MTHFR* C677T illustrates this more sharply: the T/T risk genotype prevalent in European and Asian POAG patients is entirely absent in Saudis, with the C/T genotype instead conferring risk, a complete allelic redistribution that invalidates direct extrapolation of effect sizes across ancestries. *APOE* ε2 prese1nts the starkest discordance: it drives a near five-fold PACG risk in Saudi cohorts (OR = 4.82) yet shows inconsistent or null effects in the UK Biobank and large international datasets, suggesting either genuine population-specific enrichment or that global studies lack statistical power in this subgroup. For PXG, *LOXL1* risk alleles (rs1048661-G, rs3825942-G) replicate in Saudis as expected from the landmark Icelandic GWAS (Thorleifsson et al., 2007) [53], but two additional non-synonymous variants (D4w84E, Y559D) found exclusively in Saudi PXG patients are absent from all global datasets, pointing to population-private functional variation the field has not characterised. *CYP1B1*, the dominant causal gene in Saudi JOAG and PCG, is essentially invisible in global GWAS because consanguinity-amplified monogenic signals are structurally underpowered in standard case–control GWAS designs built for common variant discovery. Multiple loci with established POAG signals elsewhere, including *GAS7*, *TMCO1*, *CDKN2B*, *ATOH7*, and *ABCA1*, failed entirely in Saudi cohorts, further confirming that polygenic risk scores derived from European or East Asian GWAS will systematically misestimate risk in this population. *ACVR1* (rs12997) runs in the opposite direction: it has no prominent profile in large GWAS databases yet shows consistent, cross-subtype associations across Saudi POAG, PACG, and PXG, marking it as a candidate population-enriched signal that warrants targeted replication and functional follow-up. The bottom line is that Saudi glaucoma genetics partially overlaps with global GWAS findings at one locus (*SIX1/SIX6*) and diverges at nearly every other, making locally anchored GWAS and population-specific polygenic risk modelling an urgent priority rather than an optional refinement.

Narta et al. (2023) [54], performed WES on 31 individuals from nine *MYOC*-negative Indian familial glaucoma families, five POAG and four PACG, validating prioritized variants in an independent cohort of 960 cases and 576 controls. The two-tier strategy identified seven novel candidate genes: *AQP5*, *SRFBP1*, *CDH6*, and *FOXM1* in POAG, and *ACACB*, *RGL3*, and *LAMA2* in PACG, all carrying rare deleterious SNVs exclusive to glaucoma cases. *SRFBP1* is particularly significant as it maps within the GLC1M juvenile-onset POAG linkage locus on chromosome 5q, providing a long-awaited candidate for a locus mapped over a decade ago without a confirmed causal gene. Pathway analysis converged on ECM organization for both subtypes and lipid metabolism specifically for PACG, with one family carrying co-segregating variants in *LDLR*, *ACACB*, and *PCSK9* alongside hypercholesterolemia, mechanistically linking cholesterol dysregulation to angle-closure pathology. Single-cell transcriptomic analysis placed candidate gene expression in retinal ganglion cells, corneal epithelial cells, and Schwalbe’s line cells, providing tissue-level biological plausibility absent from purely associative studies. The contrast with the Saudi review is fundamental: all 33 Saudi studies relied on candidate-gene Sanger sequencing or targeted SNP genotyping, and no family-based WES or WGS has been performed in any Saudi glaucoma cohort. Narta et al. (2023) [54], demonstrates exactly what that absence costs: known Mendelian genes explained none of the nine Indian families, yet WES resolved novel candidates where targeted approaches would have returned empty results, directly paralleling the unsolved *CYP1B1*-negative Saudi PCG fraction. Pathway-level findings show meaningful overlap between populations: ECM remodeling is implicated in both through different genes (*LAMA2*, *CDH6* in Indians; *SMOC2*, *LOXL1* in Saudis), and lipid metabolism emerges in PACG in both (*LDLR*/*PCSK9*/*ACACB* in Indians; *APOE* ε2 in Saudis), strengthening the case that cholesterol dysregulation is a genuine cross-population PACG mechanism. *RGL3*’s role in regulating NF-κB and autophagy through the OPTN-TBK1 axis identified in the Indian study maps directly onto the neuroinflammatory pathway the Saudi review acknowledges but leaves mechanistically uncharacterised. Saudi Arabia’s consanguinity rate of 56%, exceeding that of the outbred Indian families studied here, would structurally compress the homozygous rare variant search space even further, making WES in Saudi familial glaucoma pedigrees likely to yield comparable or richer discovery. The immediate priority for the Saudi glaucoma genetics program is applying an identical family-based WES strategy to *CYP1B1*-negative PCG trios and *MYOC*-negative adult-onset pedigrees, combined with the single-cell expression and pathway validation framework demonstrated here, to convert the current SNP catalogue into a mechanistically grounded gene map.

### 3.2. Pathophysiological Insights

Genetic associations observed in Saudi cohorts investigate biological pathways that potentially link genotype to glaucoma clinical phenotypes. The following are the observed associations which implicate specific biological pathways:

- Oxidative stress: The *SOD2* rs4880 variant association with PACG severity suggests reduced mitochondrial antioxidant capacity increases optic nerve vulnerability [29]. Additionally, *FNDC3B* polymorphisms and other extracellular matrix genes suggest that disturbed matrix turnover could play a significant role in trabecular meshwork stiffness and optic nerve head resilience [32].
- Growth Factor Signaling: Disruption of growth factor signaling provides a unifying explanation for genetic effects that are observed across glaucoma subtypes. The *ACVR1* rs12997 associations across POAG, PACG, and PXG implicate the BMP/TGF-β signaling pathways in trabecular meshwork (TM) stiffness and optic nerve resilience [25,27]. A plausible molecular mechanism here is that variations in the 3′ UTR may affect how stable the transcripts are or influence their interaction with microRNAs, which in turn could adjust receptor levels and the subsequent SMAD/Wnt signaling pathway.
- Extracellular Matrix (ECM)*:* The polymorphisms in *SMOC2* (specifically the GA genotype) have been associated with PACG. This suggests that changes in collagen production and matrix metalloproteinase activity could drive angle-closure development, while the protective G/G genotype points to how specific alleles can influence extracellular matrix balance. In the case of PXG, risk alleles for *LOXL1*, along with unique non-synonymous variants specific to Saudi populations (D484E, Y559D), highlight issues with elastogenesis and deposition of fibrillar materials as significant contributors to pseudoexfoliation-related pathology [31]. There are also intersections with metabolic and regulatory pathways. The *MTHFR* C677T genotype in Saudi individuals, especially the prevalence of C/T genotypes in POAG patients and the rarity of T/T homozygotes, suggests that even small reductions in folate-dependent methylation can lead to hyperhomocysteinemia. This may enhance vascular problems, alter the matrix, and increase susceptibility in the optic nerve [24].
- Epigenetic Regulation: The protective effect of *DICER1* rs3742330 variant and *DICER1/DROSHA* GA haplotype suggests that microRNA biogenesis may modulate the expression of genes involved in ECM homeostasis [30]. In the Saudi glaucoma patients investigated, epigenetic signal is limited to the *DICER1* rs3742330 protective association in PXG (OR = 0.38), where the G allele and the *DICER1/DROSHA* G/A haplotype collectively implicate microRNA biogenesis as a modulator of ECM homeostasis, likely through post-transcriptional suppression of *LOXL1* and matrix metalloproteinase expression that would otherwise drive fibrillar material accumulation. The manuscript also notes that *LOXL1* risk penetrance is incomplete even among homozygous carriers, and attributes this to supplementary epigenetic and environmental factors including RNA methylation, though without specifying mechanism. *MTHFR* C677T adds a metabolic–epigenetic bridge: the C/T genotype prevalent in Saudi POAG patients reduces folate-dependent methylation capacity, elevating homocysteine and globally hypomethylating genomic loci, which can dysregulate trabecular meshwork gene expression and amplify oxidative optic nerve injury. The *SMOC2* PACG association further intersects with epigenetic regulation, as SMOC2 protein interacts with microRNA-mediated suppression of SMAD/Wnt pathway components, linking an SNP-level genetic signal to a post-transcriptional regulatory layer.

Lastly, the fact that pathogenic *MYOC* variants are absent in Saudi cohorts with JOAG and PCG, despite *MYOC’s* known significance in POAG among other groups, highlights the need to consider population-specific factors in understanding the genetic underpinnings of these diseases [14]. Overall, the findings from Saudi data suggest that interactions among extracellular matrix remodeling, oxidative stress, vascular issues, and disrupted growth-factor signaling play crucial roles in determining the susceptibility to glaucoma, its various subtypes, and the severity of clinical outcomes. *MYOC* mutations are absent from Saudi JOAG and PCG cohorts, but the finding rests on a single study of only 35 JOAG patients, a sample too small to reliably exclude a gene contributing at 3–4% frequency. Globally, *MYOC* is the most frequently mutated gene in POAG/JOAG, accounting for 2–4% of POAG and up to 33% of JOAG cases in some cohorts, with the specific mutation profile varying sharply by ancestry: Gln368Stop dominates in Europeans, Pro370Leu drives a founder-effect JOAG in Chinese families, and Gln48His is exclusive to Indian patients. The structural explanation for Saudi absence is straightforward: when *CYP1B1* occupies 85.7% of solved JOAG cases, the residual unsolved fraction is too small to detect *MYOC* at its expected background frequency. A further confounder is the dominant-recessive dichotomy: *MYOC* acts through autosomal dominant gain-of-toxic-function in trabecular meshwork cells, a mechanism that does not benefit from the homozygosity amplification that high consanguinity provides for recessive *CYP1B1*, making dominant *MYOC* families less likely to be recruited in studies built around consanguineous pedigrees. The parallel observation that Gln368Stop is also absent in Indian cohorts suggests this allele is genuinely rare across non-European ancestries, not uniquely absent in Saudis. Collectively, the evidence points to a combination of true low allele frequency, ascertainment bias, and insufficient sample size rather than a biologically meaningful absence. A systematic screen of at least 300–500 *CYP1B1*-negative Saudi JOAG patients may be required before absence of *MYOC* can be stated with any confidence.

## 4. Limitations

The existing Saudi glaucoma literature has several limitations. Many studies, especially those on juvenile-onset cohorts, are small, which reduces statistical power and limits how confidently findings can be generalized beyond the sampled families or centers. Most reports are observational case–control analyses and stop at association; few include functional experiments to show how specific variants alter gene expression, protein function, or cellular behavior. Additionally, environmental and systemic modifiers remain under-studied: diet, folate status, smoking, ultraviolet exposure, and vascular comorbidities have not been systematically incorporated into genetic models, yet they plausibly interact with oxidative stress, matrix, and vascular pathways implicated by the genetic data. Addressing these gaps will require larger, multicenter cohorts with harmonized phenotyping, family-based sequencing to refine penetrance estimates, and targeted functional studies that test variant effects in relevant ocular tissues. The most critical gap in Saudi glaucoma genetics is the complete absence of WES or WGS data across all 33 reviewed studies, which relied exclusively on Sanger sequencing of candidate genes or targeted SNP genotyping. This is particularly indefensible given that consanguineous populations are structurally ideal for WES-based discovery: high background homozygosity compresses the variant search space, enriches rare recessive alleles to detectable frequencies, and simplifies segregation analysis in family trios. Approximately 15–24% of Saudi PCG patients carry no pathogenic *CYP1B1* variant, and the manuscript lists candidate genes from this unsolved fraction (*SLC4A4*, *SLC4A11*, *CPAMD8*, *KERA*, *ADAM9*, *BCO2*, *TULP2*, *DGKQ*) without functional validation or independent replication. The global WES literature has already validated several of these: biallelic *CPAMD8* variants are now an established cause of childhood and juvenile open-angle glaucoma, and *FOXC1* accounts for approximately 6% of *CYP1B1*-negative PCG cases, neither finding reflected in any Saudi study. WES in South African PCG children identified pathogenic *TEK* variants disrupting Schlemm’s canal development and putatively damaging rare variants in 12 additional children, demonstrating that unbiased exome sequencing substantially outperforms targeted Sanger approaches in resolving the unsolved fraction. WES in *CYP1B1*-negative Pakistani PCG families, a high-consanguinity population directly comparable to Saudi Arabia, identified novel pathogenic *LTBP2* and *PXDN* variants missed entirely by prior Sanger screening. For adult disease, a WES study of 110,260 UK Biobank participants identified 40 novel rare-variant IOP genes, including *BOD1L1*, *ACAD10*, and *HLA-B*, with six, including *MTOR*, *EGLN2*, and *ADRB1*, representing druggable targets, none detectable by the SNP approaches used in Saudi cohorts. A subsequent WGS study of 488,888 individuals confirmed known genes and identified over 68 previously unreported glaucoma genes including *FMO4*, *ANGPTL7*, and *AREL1*, expanding the biological landscape well beyond what common-variant GWAS captured. Several pathways already implicated by Saudi SNP data, specifically BMP/TGF-β signaling via *ACVR1* and ECM remodeling via *SMOC2* and *LOXL1*, carry rare coding variants detectable only by sequencing that remain entirely uncharacterized in this population. The TEK-angiopoietin pathway, central to Schlemm’s canal development and now established in multiple non-Saudi PCG WES studies, has never been systematically screened in the Saudi *CYP1B1*-negative fraction. The current genetic studies on the Saudi glaucoma patients is dominated by *CYP1B1* in early-onset disease and modest-effect SNPs in adults, almost certainly underestimates rare variant contributions across all subtypes. The immediate practical priority will be a family-based WES in 100–200 *CYP1B1*-negative Saudi PCG trios and rare-variant burden testing in 500+ adult POAG and PACG cases, which would convert the current candidate-SNP catalogue into a mechanistically grounded genetic map suitable for clinical panel development and therapeutic target prioritization.

## 5. Clinical and Public Health Implications

### 5.1. Genetic Screening Programs

Offer targeted testing where it will change management. Prioritize *CYP1B1* testing for infants and children with congenital or juvenile-onset glaucoma and for first-degree relatives in affected families; pair testing with structured pre- and post-test genetic counselling. For adult patients, consider research-grade panels that include locally relevant loci (for example, *LOXL1*, *APOE*, *NOS3*, *ACVR1*) in high-risk clinics, but avoid population-wide screening until larger replication studies and cost-effectiveness data are available. Pilot cascade-testing programs in regions with high PCG prevalence to measure uptake, diagnostic yield, and family outcomes before broader rollout.

### 5.2. Personalized Therapies

Genetic information can inform hypothesis-driven interventions. Patients carrying *NOS3* risk alleles are plausible candidates for trials of nitric-oxide-donor therapies or other vascular-targeted approaches aimed at improving optic nerve perfusion. For *APOE* ε2 carriers with PACG, adjunctive studies of vascular-modifying or lipid-lowering strategies may be warranted. These are investigational pathways: clinical adoption should follow randomized evaluation demonstrating safety and benefit.

### 5.3. Public Health Awareness Campaigns

Public health messaging should emphasize early detection and practical actions. Integrate targeted glaucoma screening into primary-care encounters for families with known early-onset disease, and develop culturally appropriate educational materials about genetic risk and available counselling services. Campaigns should focus on actionable steps—screening, timely referral, and family testing—rather than deterministic messaging about genetics.

## 6. Future Directions

### 6.1. Research Priorities

Large, well-powered genetic studies are the immediate priority. Saudi research should move beyond small candidate-gene series to population-scale genome-wide association studies and well-designed sequencing projects that include family-based cohorts. Parallel efforts must establish deeply phenotyped case registries and biobanks with standardized clinical metrics so that genotype–phenotype correlations and penetrance estimates can be robustly derived. Functional follow-up is equally important: targeted experiments (for example, allele-specific expression, reporter assays, and cell models) should test the biological consequences of prioritized variants, and focused use of genome editing in ocular cell systems can validate causal mechanisms for genes such as *ACVR1* and *SMOC2*.

### 6.2. Technological Advancements

Develop and validate polygenic risk scores tailored to the Saudi population rather than importing scores derived from other ancestries. Combine genetic risk estimates with quantitative imaging, intraocular pressure trajectories, and clinical covariates using transparent machine-learning frameworks to improve individual risk stratification. Pilot implementations should evaluate clinical utility, cost-effectiveness, and equity before any routine deployment.

### 6.3. Collaborative Frameworks

Maximizing impact requires coordinated, multicenter collaboration. Form a national consortium that links tertiary eye centers, genetics laboratories, and public-health agencies; harmonize protocols for recruitment, phenotyping, and data sharing; and create governance structures for ethical data use. Regional partnerships across the Gulf Cooperation Council and participation in international consortia (for example, NEIGHBOR and the International Glaucoma Genetics Consortium) will increase power for discovery and help distinguish universal from population-specific loci.

### 6.4. Proposal of National Program for PCG Eradication

Because ~90% of PCG-cases are hereditary with autosomal recessive inheritance, many affected children are born to unaffected carrier parents, especially in consanguineous marriages. *CYP1B1* mutations explain most Saudi PCG, and just four mutations may cover >90% of cases, making a focused, relatively low-cost premarital test realistic. Authors propose a national premarital screening and genetic counseling program, similar to the existing hemoglobinopathy program, with an arm for high-risk families and another for broader screening where burden is highest. Economically, they authors argue that screening could beat treatment costs by a wide margin, and, paired with counseling, could prevent future cases and move Saudi Arabia toward eradicating PCG-related childhood blindness [55].

## 7. Conclusions

The evidence reviewed here indicates that glaucoma in Saudi Arabia reflects a distinct genetic landscape shaped by high consanguinity, founder alleles, and population-specific variant frequencies. *CYP1B1* stands out as the principal causal gene for congenital and juvenile disease, while loci such as *LOXL1*, *APOE*, *NOS3*, and *ACVR1* emerge as important contributors to adult-onset and pseudoexfoliation forms. Many associations remain provisional, however, and translating these findings into clinical practice will require larger, well-phenotyped cohorts, functional validation of candidate variants, and studies that integrate environmental and systemic modifiers. Building national registries, harmonizing phenotyping, and joining international consortia will accelerate replication and enable the development of targeted screening, counselling, and intervention strategies tailored to the Saudi population.

## Figures and Tables

**Table 1:** Glaucoma-Associated Genes and Variants across Subtypes in the Saudi Population.

Gene	Disease	Amino Acid Change	Zygosity	Phenotypic Effect	Penetrance	Refs. No.
*CYP1B1*	JOAG/PCG	p.G61E	Homozygous/Compound heterozygous	High IOP, bilateral, advanced cupping	87.70%	[14,16,17]
		p.R469W	Homozygous/Compound heterozygous	Severe early-onset disease	93%	[16,17,18]
		p.E229K	Heterozygous	Rare novel variant	Unknown	[16,19]
		p.L432V	Heterozygous/Compound heterozygous	Variable severity	Variable	[14,19]
	PCG	p.H279L	Homozygous/Heterozygous	Early onset, severe	High	[18]
		p.R355X				
		p.R368H	Homozygous	Likely benign, low penetrance	23%	[17]
*MYOC*	JOAG	No pathogenic variants identified	-	-	-	[14]
*LTBP2*			-	-	-	[14]

**Table 2:** Genetic Risk Factors (SNPs) Associated with Glaucoma Subtypes in the Saudi Population.

Disease	Gene	Chr	Genomic Location	SNP ID	Risk Allele/ Genotype	Patients (n)	Controls (n)	Odds Ratio (OR)	95% CI	*p* Value	Ref. No.
POAG	*SIX1/SIX6*	14	Intergenic	rs10483727	T	92	94	1.7	1.11–2.58	0.013	[22]
	*NOS3*	7	Exon 7	rs1799983	T	94 (Men)	98 (Men)	1.77	1.07–2.94	0.025	[23]
	*MTHFR*	1	Exon 4	C677T	C/T	210	280	1.6	#	0.02	[24]
	*ACVR1*	2	3′ UTR	rs12997	G	150	250	1.39	1.03–1.87	0.027	[25]
PACG	*APOE*	19	Exon 4	rs429358	ε2 Carriers	92	251	4.82	1.52–15.26	0.007	[26]
	*ACVR1*	2	3′ UTR	rs12997	G/G	101	250	>2.0	#	<0.05	[27]
	*SMOC2*	6	Intronic	rs13208776	G/A	64	202	2.16	1.13–4.13	0.04	[28]
	*SOD2*	6	Exon 2	rs4880	C	139	403	#	#	0.391 ●	[29]
PXG	*DICER1*	14	3′ UTR	rs3742330	G	94	246	0.38 *●	0.16–0.92	0.017	[30]
	*ACVR1*	2	3′ UTR	rs12997	G/G	95	250	2.04	0.99–4.18	0.049	[27]
	*LOXL1*	15	Exon 1	rs1048661	G	93	101	#	#	0.0056	[31]
	*LOXL1*	15	Exon 1	rs3825942	G	93	101	#	#	0.000005	[31]
	*FNDC3B*	3	Intronic	rs7636836	T	94	246	2.69	1.11–6.51	0.029	[32]

# Data not reported in the original study. ● Statistically non-significant for disease susceptibility (*p* > 0.05); however, the variant showed a significant association with clinical severity indices. * Odds Ratio (OR) < 1.0 indicates a protective effect; the presence of this allele/genotype significantly reduces the risk of developing the disease.

## Data Availability

No new data were created. Data sharing is not applicable to this article.

## Abbreviations

The following abbreviations are used in this manuscript:

ACVR1	Activin A Receptor Type 1
APOE	Apolipoprotein E
BMP	Bone Morphogenetic Protein
CYP1B1	Cytochrome P450 Family 1 Subfamily B Member 1
DICER1	Dicer 1, Ribonuclease III
DROSHA	Drosha Ribonuclease III
GWAS	Genome-Wide Association Study
IOP	Intraocular Pressure
JOAG	Juvenile-Onset Open-Angle Glaucoma
LOXL1	Lysyl Oxidase Like 1
MeSH	Medical Subject Headings
MTHFR	Methylenetetrahydrofolate Reductase
NOS3	Nitric Oxide Synthase 3
PACG	Primary Angle-Closure Glaucoma
PCG	Primary Congenital Glaucoma
PEG	Pseudoexfoliation Glaucoma
POAG	Primary Open-Angle Glaucoma
PXG	Pseudoexfoliation Glaucoma
SIX1/SIX6	SIX Homeobox 1/SIX Homeobox 6
SMOC2	SPARC Related Modular Calcium Binding 2
SNP	Single Nucleotide Polymorphism
SOD2	Superoxide Dismutase 2
TGF-β	Transforming Growth Factor-Beta
